# Supplementary material for: Dietary patterns; serum concentrations of selenium, copper, and zinc; copper/zinc ratio; and total antioxidant status in patients with glaucoma
Source: PLoS One. 2024 Apr 2;19(4):e0301511. doi: 10.1371/journal.pone.0301511 (PMC10986966; doi:10.1371/journal.pone.0301511)
Supplement: S1 Table — (DOCX) [file pone.0301511.s001.docx]

**Supplementary Table 1. Relationships between frequency of food product consumption and serum Se, Cu, Zn, and TAS levels according to multiple stepwise regression analysis.**

| **Independent variables** | **β Coefficient (SE)** | | | **Significance level** | | **Adjusted R^2^** |
| --- | --- | --- | --- | --- | --- | --- |
| **Selenium** | | | | | | |
| 1. Fish 2. Meat 3. Coffee 4. Milk 5. Eggs 6. Butter 7. Beer 8. Bacon, lard 9. White bread 10. Potatoes 11. Cheese 12. Sweet bread 13. Tea 14. Legumes 15. Vegetable oil | | 0.438 (0.090)  0.354 (0.107)  0.236 (0.087)  0.206 (0.087)  0.197 (0.086)  0.163 (0.096)  -0.291 (0.097)  -0.285 (0.110)  -0.219 (0.093)  -0.232 (0.103)  -0.149 (0.093)  -0.162 (0.103)  -0.123 (0.092)  -0.112 (0.088)  -0.110 (0.091) | 0.0001*  0.002*  0.009*  0.021*  0.026*  0.095  0.004*  0.012*  0.023*  0.028*  0.117  0.122  0.188  0.208  0.230 | | 0.50 | |
| **Zinc** | | | | | | |
| 1. Ham 2. Offal 3. Potatoes 4. Poultry 5. Coffee 6. Meat 7. Vegetable oil 8. Raw vegetables 9. White cheese 10. Beer 11. Tinned meat 12. Margarine 13. Fish 14. Sweet drinks 15. Jam | | 0.326 (0.093)  0.234 (0.092)  0.224 (0.103)  0.185 (0.096)  0.153 (0.097)  0.144 (0.104)  -0.356 (0.094)  -0.218 (0.092)  -0.187 (0.094)  -0.183 (0.101)  -0.159 (0.099)  -0.135 (0.091)  -0.127 (0.104)  -0.104 (0.094)  -0.098 (0.095) | 0.0001*  0.014*  0.034*  0.057  0.120  0.174  0.0003*  0.021*  0.050  0.075  0.115  0.143  0.225  0.272  0.306 | | 0.42 | |
| **Copper** | | | | | | |
| 1. Wholegrain bread 2. Fruits 3. Coffee 4. Eggs 5. Sugar 6. Tinned fish 7. Legumes 8. Jam 9. Bacon, lard 10. Potatoes 11. Grits, rice 12. Vegetable oil 13. Raw vegetables 14. Cheese 15. Jam | | 0.289 (0.105)  0.220 (0.098)  0.186 (0.104)  0.170 (0.094)  0.164 (0.106)  0.161 (0.110)  0.140 (0.100)  0.117 (0.101)  -0.348 (0.111)  -0.337 (0.120)  -0.240 (0.112)  -0.180 (0.102)  -0.140 (0.100)  -0.140 (0.102)  -0.123 (0.101) | 0.008*  0.028*  0.078  0.078  0.128  0.130  0.168  0.251  0.003*  0.006*  0.038*  0.850  0.165  0.177  0.22 | | 0.38 | |
| **TAS** | | | | | | |
| 1. Potatoes 2. Cheese 3. Offal 4. Legumes 5. Ham 6. Vegetable oil 7. Margarine 8. Fish 9. Butter 10. Jam 11. Canned meat 12. Tea | | 0.227 (0.118)  0.186 (0.113)  0.172 (0.109)  0.166 (0.108)  0.157 (0.114)  0.062 (0.120)  -0.318 (0.122)  -0.208 (0.116)  -0.173 (0.123)  -0.144 (0.114)  -0.143 (0.114)  -0.120 (0.114) | 0.060  0.104  0.120  0.130  0.173  0.604  0.012*  0.078  0.164  0.210  0.213  0.294 | | 0.13 | |

TAS, total antioxidant status
