# Supplementary material for: Dietary patterns; serum concentrations of selenium, copper, and zinc; copper/zinc ratio; and total antioxidant status in patients with glaucoma
Source: PLoS One. 2024 Apr 2;19(4):e0301511. doi: 10.1371/journal.pone.0301511 (PMC10986966; doi:10.1371/journal.pone.0301511)
Supplement: S2 File — (DOCX) [file pone.0301511.s003.docx]

Opracowanie statystyczne

| **Klient:** | Joanna Konopińska |
| --- | --- |
| **Autor:** | [www.statystykadlaciebie.pl](http://www.statystykadlaciebie.pl)  [marta.nowak@statystykadlaciebie.pl](mailto:marta.nowak@statystykadlaciebie.pl) |
| **Podstawa opracowania:** | Dane dostarczone przez Klienta |
| **Data:** | Czerwiec 2023 |

# Analiza statystyczna

*opis metodologii analizy statystycznej, do wykorzystania w rozdziale o metodologii*

The analysis was run in R statistical software (version 4.1.2). Nominal variables were described with number of patients and their % share in respective group. Numerical variables were described with mean and standard deviation as well as median and interquartile range. Normality of distribution was preliminarily tested with Shapiro-Wilk test and further verified with skewness and kurtosis. Levene test was used to assess variance homogeneity. Groups comparisons were performed with Pearson chi-square test, Fisher exact test, t-Student independent test, t-Welch independent test, Mann-Whitney U test, Anova and Anova Welch analyses and Kruskal-Wallis test, as appropriate. Relationships between two numerical variables were assessed with Spearman correlation. Significance level of *α* = 0.05 was assumed for all analyses.

# Wyniki

**Demographic characteristics of study groups**

The analysis was based on two groups of patients: with glaucoma (N = 109) and control group (N = 65). Proportion of males was 32.4% and 23.1% in glaucoma group and control group, respectively, with no statistical difference between groups, p = 0.260. Mean age in glaucoma group was 70.70 ± 10.63 years and in control group it was 52.89 ± 11.82 years. There was significant difference in age between groups, MD = 19.00 CI_95_ [14.00;22.00], p < 0.001. BMI was available only for patients with glaucoma and was equal to 28.46 ± 4.42 kg/m^2^, table 1.

Table 1. Demographic characteristics of study groups

| **Variable** | **Glaucoma** | | **Control** | | **MD (95% CI)** | **p** |
| --- | --- | --- | --- | --- | --- | --- |
|  | **n (%) / Mean ± SD** | **Median (Q1;Q3)** | **n (%) / Mean ± SD** | **Median (Q1;Q3)** |  |  |
| N | 108 (100.0) | - | 65 (100.0) | - | - | - |
| Sex, male | 34 (32.4) | - | 15 (23.1) | - | - | 0.260 |
| Age, years | 70.70 ± 10.63 | 73.00 (66.00;77.00) | 52.89 ± 11.82 | 54.00 (45.00;60.00) | 19.00 (14.00;22.00) | **< 0.001** |
| BMI, kg/m^2^ | 28.46 ± 4.42 | 27.73 (25.39;31.04) | - | - | - | **-** |

**Notes:** SD – standard deviation, Q1 – first quartile, Q3 – third quartile, MD – median difference (glaucoma vs control). Data presented as n (%) for sex and mean ± standard deviation and median (Q1;Q3) for age and BMI. Comparisons performed with Mann-Whitney U test (age) and Pearsona chi-square test (sex).

**Comparison of Se, Zn, Cu, Cu/Zn and TAS between study groups**

Groups differed significantly with levels of Se, Zn and Cu/Zn. Levels of Se and Zn were lower among glaucoma patients compared to control group, MD = -8.82 CI_95_ [-14.14;-4.42], p < 0.001 and MD = -0.06 CI_95_ [-0.13;-0.04], p < 0.001. Cu/Zn was higher among glaucoma patients compared to control group, MD = 0.17 CI_95_ [0.06;0.36], p = 0.006. Levels of Cu and TAS did not vary between groups from statistical perspective, table 2. Visualization of the level of chemical elements in both groups was presented in figure 1.

Table 2. Comparison of Se, Zn, Cu, Cu/Zn and TAS between study groups

| **Variable** | **Glaucoma** | | **Control** | | **MD (95% CI)** | **p** |
| --- | --- | --- | --- | --- | --- | --- |
|  | **Mean ± SD** | **Median (Q1;Q3)** | **Mean ± SD** | **Median (Q1;Q3)** |  |  |
| Se, mg/L | 68.80 ± 16.89 | 66.66 (58.32;78.28) | 86.02 ± 50.04 | 75.48 (69.07;86.87) | -8.82 (-14.14;-4.42) | **< 0.001** |
| Zn, mg/L | 0.76 ± 0.12 | 0.76 (0.68;0.82) | 0.85 ± 0.16 | 0.82 (0.75;0.94) | -0.06 (-0.13;-0.04) | **< 0.001** |
| Cu, mg/L | 1.10 ± 0.34 | 1.09 (0.89;1.29) | 1.08 ± 0.37 | 0.99 (0.85;1.24) | 0.10 (-0.05;0.16) | 0.229 |
| Cu/Zn, mmol | 1.52 ± 0.52 | 1.47 (1.15;1.82) | 1.35 ± 0.57 | 1.29 (0.98;1.45) | 0.17 (0.06;0.36) | **0.006** |
| TAS, mmol/L | 1.51 ± 0.28 | 1.55 (1.41;1.65) | 1.50 ± 0.58 | 1.50 (1.07;1.77) | 0.05 (-0.08;0.20) | 0.416 |

**Notes:** SD – standard deviation, Q1 – first quartile, Q3 – third quartile, MD – median difference (glaucoma vs control). Comparisons performed with Mann-Whitney U test.

**

Figure 1. Distribution of chemical elements by study group

**Comparison of norms for Se, Zn, Cu and TAS between study groups**

There was a significant difference between groups in proportion of patients with Se, Zn and TAS below norm/in norm/above norm (p < 0.001, p = 0.014 and p < 0.001, respectively). In case of Se the proportion of patients with its level in norm was 46.7% and 75.4% in glaucoma and control groups, respectively. There was higher proportion of patients with Se below norm in glaucoma group compared to control group (49.5% vs 15.4%). Number of patients with Se above norm was lower in glaucoma group (3.8%, n = 4 vs 9.2%, n = 6). Proportion of patients with Zn in norm was lower within patients with glaucoma (64.8% vs 80.0% in control group). There was higher proportion of patients with Zn below norm in glaucoma group compared to control group (35.2% vs 18.5%). No patients had Zn above norm in glaucoma group and in case of one patient the level of Zn was exceeded above norm in control group. Proportion of patients with TAS in norm was over two times higher in glaucoma group compared to control group (69.5% vs 30.4%). There was lower proportion of patients with TAS below norm in glaucoma group compared to control group (18.1% vs 48.2%). Number of patients with TAS above norm was also lower in glaucoma group (12.4% vs 21.4% in control group). No statistical difference of patients below norm/in norm/above norm for Cu was confirmed, table 3.

Table 3. Comparison of Se, Zn, Cu and TAS between study groups

| **Variable** | **Glaucoma** | **Control** | **p** |
| --- | --- | --- | --- |
| **Se** |  |  |  |
| Low | 52 (49.5) | 10 (15.4) | **< 0.001** |
| Normal | 49 (46.7) | 49 (75.4) |  |
| High | 4 (3.8) | 6 (9.2) |  |
| **Zn** |  |  |  |
| Low | 37 (35.2) | 12 (18.5) | **0.014^1^** |
| Normal | 68 (64.8) | 52 (80.0) |  |
| High | 0 (0.0) | 1 (1.5) |  |
| **Cu** |  |  |  |
| Low | 14 (13.3) | 4 (6.2) | 0.312 |
| Normal | 85 (81.0) | 56 (86.2) |  |
| High | 6 (5.7) | 5 (7.7) |  |
| **TAS** |  |  |  |
| Low | 19 (18.1) | 27 (48.2) | **< 0.001** |
| Normal | 73 (69.5) | 17 (30.4) |  |
| High | 13 (12.4) | 12 (21.4) |  |

**Notes:** Comparisons performed with Pearson chi-square teat or Fisher exact test^1^.

**Relationship between level of chemical elements and RNFL, BCVA, IOP, BMI, type of glaucoma as well as sex, within patients with glaucoma**

Significance of the relationship between level of chemical elements and RNFL, BCVA, IOP was measured with Spearman correlation analysis. Significant correlations were identified between Se and RNFL as well as Cu and RNFL, p = 0.033 and p = 0.047, respectively. Strength of the relationship between Se and RNFL measured with rho correlation coefficient was 0.22, which indicated positive relationship with weak strength. Strength of the relationship between Cu and RNFL was 0.20, which indicated positive yet weak relationship. Correlation between Cu/Zn and IOP was also statistically significant, p = 0.024. Strength of the relationship was -0.22, which indicated negative and weak relationship. Other assessed relationships were not significant, table 4. Visualization of significant correlations was presented as figure 2.

Table 4. Correlation of Se, Zn, Cu, Cu/Zn and TAS with optical parameters and BMI

| **Variable** | **RNFL, μm** | | **BCVA** | | **IOP, mmHg** | | **BMI, kg/m^2^** | |
| --- | --- | --- | --- | --- | --- | --- | --- | --- |
|  | **rho** | **p** | **rho** | **p** | **rho** | **p** | **rho** | **p** |
| Se, mg/L | **0.22** | **0.033** | 0.13 | 0.180 | -0.06 | 0.580 | 0.03 | 0.758 |
| Zn, mg/L | 0.12 | 0.225 | -0.05 | 0.638 | 0.16 | 0.114 | 0.00 | 0.998 |
| Cu, mg/L | **0.20** | **0.047** | 0.07 | 0.477 | -0.18 | 0.065 | 0.02 | 0.876 |
| Cu/Zn, mmol | 0.12 | 0.239 | 0.05 | 0.598 | **-0.22** | **0.024** | 0.01 | 0.911 |
| TAS, mmol/L | -0.15 | 0.155 | -0.13 | 0.189 | 0.10 | 0.330 | -0.10 | 0.314 |

**Notes:** rho – Spearman correlation coefficient.

**

Figure 2. Significant relationships between chemical elements and optical parameters (Se and RNFL, Cu and RNFL, Cu/Zn and IOP) within patients with glaucoma

No relationship between chemical elements and types of glaucoma was confirmed to be significant (p > 0.05), table 5.

Table 5. Comparison of Se, Zn, Cu, Cu/Zn and TAS between types of glaucoma

| **Variable** | **JPOK (n = 58)** | | **JPEX (n = 16)** | | **JZK (n = 17)** | | **Other types (n = 7)** | | **p** |
| --- | --- | --- | --- | --- | --- | --- | --- | --- | --- |
|  | **Mean ± SD** | **Median (Q1;Q3)** | **Mean ± SD** | **Median (Q1;Q3)** | **Mean ± SD** | **Median (Q1;Q3)** | **Mean ± SD** | **Median (Q1;Q3)** |  |
| Se, mg/L | 66.44 ± 17.37 | 63.74 (55.42;75.97) | 76.40 ± 18.53 | 71.05 (63.23;81.46) | 72.73 ± 17.14 | 72.81 (59.88;83.02) | 67.63 ± 11.38 | 66.91 (59.78;76.19) | 0.175 |
| Zn, mg/L | 0.77 ± 0.14 | 0.76 (0.68;0.85) | 0.74 ± 0.09 | 0.77 (0.70;0.81) | 0.76 ± 0.09 | 0.76 (0.71;0.82) | 0.77 ± 0.11 | 0.75 (0.71;0.82) | 0.912 |
| Cu, mg/L | 1.05 ± 0.36 | 1.02 (0.83;1.28) | 1.22 ± 0.32 | 1.17 (1.05;1.45) | 1.03 ± 0.28 | 1.07 (0.92;1.17) | 1.17 ± 0.30 | 1.16 (0.99;1.26) | 0.281 |
| Cu/Zn, mmol | 1.45 ± 0.55 | 1.38 (1.02;1.79) | 1.72 ± 0.56 | 1.61 (1.38;1.94) | 1.42 ± 0.41 | 1.47 (1.20;1.71) | 1.56 ± 0.35 | 1.67 (1.28;1.79) | 0.263 |
| TAS, mmol/L | 1.53 ± 0.25 | 1.52 (1.40;1.65) | 1.53 ± 0.28 | 1.55 (1.42;1.64) | 1.49 ± 0.35 | 1.58 (1.43;1.64) | 1.51 ± 0.17 | 1.50 (1.45;1.65) | 0.977^1^ |

**Notes:** SD – standard deviation, Q1 – first quartile, Q3 – third quartile. Comparisons performed with Anova analysis or Kruskal-Wallis test^1^.

*Typy jaskry, jak rozumiem, są nazwane za pomocą terminologii polskiej. Niestety nie znam tak szczegółowych określeń po angielsku, więc pozwolę sobie już je zostawić w takiej formie jak powyżej.*

No relationship between chemical elements and sex was confirmed to be significant (p > 0.05), table 6.

Table 6. Comparison of Se, Zn, Cu, Cu/Zn and TAS between sex of patients with glaucoma

| **Variable** | **Males (n = 34)** | | **Females (n = 71)** | | **MD (95% CI)** | **p** |
| --- | --- | --- | --- | --- | --- | --- |
|  | **Mean ± SD** | **Median (Q1;Q3)** | **Mean ± SD** | **Median (Q1;Q3)** |  |  |
| Se, mg/L | 67.70 ± 17.14 | 63.80 (56.80;75.07) | 69.32 ± 16.87 | 66.91 (59.10;79.97) | -1.62 (-8.63;5.39) | 0.648 |
| Zn, mg/L | 0.76 ± 0.11 | 0.76 (0.70;0.80) | 0.76 ± 0.13 | 0.75 (0.68;0.83) | 0.00 (-0.05;0.05) | 0.910 |
| Cu, mg/L | 1.07 ± 0.31 | 1.09 (0.88;1.26) | 1.11 ± 0.35 | 1.09 (0.89;1.32) | -0.04 (-0.18;0.10) | 0.570 |
| Cu/Zn, mmol | 1.46 ± 0.45 | 1.43 (1.11;1.76) | 1.55 ± 0.56 | 1.47 (1.16;1.84) | -0.08 (-0.30;0.13) | 0.446 |
| TAS, mmol/L | 1.57 ± 0.27 | 1.56 (1.43;1.69) | 1.49 ± 0.28 | 1.51 (1.35;1.65) | 0.05 (-0.04;0.17) | 0.304^1^ |

**Notes:** SD – standard deviation, Q1 – first quartile, Q3 – third quartile, MD – mean or median difference (males vs females). Comparisons performed with t-Student independent test or Mann-Whitney U test^1^.

**Relationship between food products and level of Se**

Level of Se was significantly related to consumption of white bread (p = 0.023), milk and milk products (p = 0.043), fresh fish (p = 0.010), eggs (p = 0.007), boiled vegetables (p = 0.032) and vitamins (p = 0.013). In case of white bread post hoc test confirmed that level of Se was significantly higher in case of patients never consuming the products compared to patients consuming them often (81.04 ± 20.18 mg/L vs 67.75 ± 15.60 mg/L). In case of milk and milk products Se was the lowest in case of patients consuming the products seldom and higher both in case of no consumption and often consumption, however post hoc test was not able to confirm significant pairwise outcome. In case of fresh fish and eggs post hoc confirmed that Se was significantly higher among patients consuming the products often, compared to patients consuming them seldom (75.75 ± 18.16 mg/L vs 65.23 ± 14.20 mg/L and 75.13 ± 17.67 mg/L vs 63.86 ± 13.41 mg/L, respectively). In case of boiled vegetables Se grew with frequency of consumption, however no specific pairwise comparison turned out to be significant in post hoc evaluation. Vitamins were taken either seldom or often with higher Se among patients with seldom intake compared to patients with often intake (76.39 ± 19.57 mg/L vs 67.01 ± 14.25 mg/L), table 7. Significant relationships were visualized on figure 3.

Table 7. Dependance of Se level (mg/L) on consumption of selected food products

| **Variable** | **Never** | **Seldom** | **Often** | **p** |
| --- | --- | --- | --- | --- |
| White bread | 81.04 ± 20.18^a^ | 75.98 ± 14.91 | 67.75 ± 15.60^a^ | **0.023** |
| Wholemeal bread | 67.74 ± 7.54 | 68.61 ± 16.93 | 72.42 ± 17.87 | 0.560 |
| Pastries | 73.81 ± 16.76 | 70.68 ± 14.75 | 69.87 ± 18.63 | 0.881 |
| Flour products | 63.72 ± 10.98 | 72.66 ± 18.61 | 69.45 ± 15.53 | 0.323 |
| Groats/rice/cereal | 59.32 ± 7.23 | 68.27 ± 16.43 | 74.17 ± 17.39 | 0.072 |
| Milk/milk beverages | 74.69 ± 19.23 | 64.20 ± 11.78 | 73.40 ± 18.20 | **0.043^3^** |
| White cheese | 71.85 ± 16.54 | 67.68 ± 15.85 | 72.62 ± 17.75 | 0.432 |
| Cheese/processed cheese | 72.85 ± 17.19 | 71.38 ± 16.76 | 68.92 ± 17.24 | 0.773 |
| Meet | 71.31 (59.45;72.38) | 66.20 (62.11;67.65) | 69.72 (58.98;83.04) | 0.368^4^ |
| Poultry | - | 66.20 (57.12;75.08) | 72.38 (61.99;82.29) | 0.088^2^ |
| Offal | 72.80 ± 15.98 | 69.67 ± 17.57 | 63.99 ± 13.32 | 0.542 |
| Sausages | 62.27 ± 11.80 | 73.95 ± 18.11 | 70.30 ± 15.94 | 0.079 |
| Premium cold cuts | 70.08 ± 22.12 | 67.44 ± 15.23 | 72.35 ± 16.92 | 0.471 |
| Cold cuts | 77.34 ± 18.53 | 72.72 ± 20.53 | 66.81 ± 12.03 | 0.127^3^ |
| Bacon/lard | 68.23 ± 13.62 | 72.81 ± 18.46 | 65.48 ± 14.50 | 0.283 |
| Meat canned food | 68.26 ± 15.61 | 71.86 ± 17.14 | 65.51 ± 22.53 | 0.565 |
| Fish canned food | 65.22 ± 16.73 | 70.62 ± 16.51 | 74.02 ± 17.43 | 0.290 |
| Fresh fish | 62.28 ± 4.58 | 65.23 ± 14.20^b^ | 75.75 ± 18.16^b^ | **0.010** |
| Eggs | 61.79 ± 10.64 | 63.86 ± 13.41^c^ | 75.13 ± 17.67^c^ | **0.007** |
| Butter | 56.65 (55.81;77.98) | 66.31 (58.71;75.30) | 71.31 (61.48;83.10) | 0.304^4^ |
| Margarine | 71.56 ± 16.23 | 69.98 ± 19.10 | 69.01 ± 12.02 | 0.874 |
| Oils | 73.56 ± 17.85 | 71.78 ± 15.31 | 69.49 ± 17.28 | 0.734 |
| Potatos | - | 73.17 (64.69;76.83) | 68.19 (58.48;80.01) | 0.387^2^ |
| Boiled vegetables | 56.83 ± 13.44 | 65.01 ± 15.03 | 73.75 ± 16.99 | **0.032** |
| Fresh vegetables | 67.57 ± 12.79 | 68.66 ± 14.64 | 71.51 ± 18.17 | 0.737 |
| Fruit | 70.55 (70.13;70.97) | 60.78 (50.22;72.60) | 69.06 (59.33;82.29) | 0.331^4^ |
| Sugar (for beverages) | 70.01 ± 14.20 | 68.23 ± 19.63 | 73.30 ± 17.79 | 0.573 |
| Jams | 71.47 ± 11.50 | 70.24 ± 19.17 | 70.15 ± 13.86 | 0.967 |
| Honey | 72.38 (63.80;83.16) | 68.48 (60.11;75.88) | 66.66 (57.27;83.04) | 0.527^4^ |
| Sweet beverages | 69.82 (62.57;82.67) | 67.90 (57.27;79.88) | 70.25 (69.40;78.89) | 0.458^4^ |
| Legumes products | 63.84 ± 16.75 | 71.52 ± 17.36 | 68.62 ± 12.93 | 0.451 |
| Beer | 70.50 ± 15.57 | 72.70 ± 18.15 | 57.20 ± 14.07 | 0.115 |
| Wine | 67.26 (61.41;75.03) | 69.71 (57.69;82.28) | - | 0.597^2^ |
| Vodka | 71.31 (62.20;81.56) | 65.36 (56.65;79.08) | - | 0.276^2^ |
| Coffee | 69.02 (64.28;74.36) | 58.68 (54.17;61.13) | 69.72 (59.33;82.29) | 0.104^4^ |
| Tea | 75.00 ± 12.93 | 68.08 ± 21.18 | 70.62 ± 16.68 | 0.767 |
| Vitamins | - | 76.39 ± 19.57 | 67.01 ± 14.25 | **0.013^1^** |

**Notes:** SD – standard deviation, Q1 – first quartile, Q3 – third quartile. Data presented as mean ± standard deviation or median (Q1;Q3), depending on normality of distribution. Comparisons performed with t-Student independent test^1^, Mann-Whitney U test^2^, Anova or Anova Welch^3^ analysis or Kruskal-Wallis test^4^. Post hoc evaluation run with Tukey test. a-c – adjusted p value produced with Tukey test (a: p = 0.030, b: p = 0.014, c: p = 0.010).

*W tabeli 7 oraz pozostałych tabelach z porównaniem poziomu parametrów ilościowych w zależności od częstości konsumpcji (tabele 7-11 i 13-17) wartość parametrów ilościowych przedstawiłam jako średnią i odchylenie standardowe lub medianę i kwartyle. Zasada jest taka, że jeżeli rozkłady danego parametru ilościowego (np. Se) są zbieżne z tzw. rozkładem normalnym (czyli spełniają odpowiedni warunek), to przedstawia się wartość jako średnią i odchylenie standardowe. Jeśli ten warunek nie jest spełniony, korzysta się z mediany i kwartyli – jest to standardowy sposób pokazywania wartości parametrów. We wcześniejszych tabelach podawałam jedno i drugie tzn. średnią oraz medianę, co też można robić i daje bardziej rozbudowany obraz, ale w tym przypadku takie podejście mocno powiększyłoby tabelę i stałaby się ona nieprzejrzysta. Gdyby jednak potrzebowała Pani kompletu informacji w tabelach, to proszę o sygnał i dołączę je.*

*Dla wina i wódki nie obserwowano pacjentów z częstą konsumpcją, nie było też nikogo w grupie never dla witamin, stąd mamy braki wartości dla tych produktów.*

*Ponadto, dla kilku innych rodzajów produktów żywnościowych analiza w tabelach 7-11 i 13-17 została przeprowadzona jedynie dla dwóch grup, czyli jedynie dla never i seldom lub seldom i often. Działo się tak w przypadkach, gdy dostępna była zbyt niska liczba obserwacji dla jakiejś częstości konsumpcji, która nie pozwoliła przeprowadzić odpowiedniego testu statystycznego dla trzech grup. Przykładowo, mamy tak dla drobiu, ponieważ brak konsumpcji drobiu został odnotowany w przypadku jednego pacjenta. W takich sytuacjach sprawdzenie zależności między konsumpcją produktu (w tym przypadku drobiu) zostało przeprowadzone dla dwóch pozostałych grup (czyli dla drobiu było to sprawdzenie czy poziom Se różnił się istotnie między pacjentami spożywającymi drób rzadko i często).*

*Testy post hoc*

*W tekście i w tabeli 7 mowa o tzw. testach post hoc – napiszę więc krótkie wyjaśnienie czym one są. Używa się ich w celu weryfikacji tego, które dokładnie pary grup odpowiadają za różnicę istotną statystycznie. Polegają one na zbadaniu każdej możliwej pary i określeniu czy ta właśnie para odpowiada za istotne zróżnicowanie poziomu parametru. Analiza post hoc jest więc pogłębieniem pierwszego kroku, czyli sprawdzenia czy średnie lub mediany są równe dla trzech grup w zależności od częstości konsumpcji czy nie są równe.*

*Czasem jest tak, że mamy istotną zależność między grupami, np. jak dla mleka w tabeli 7, a więc wiemy, że średnie poziomy Se nie były równe dla trzech grup (bo p = 0,043, czyli mniej niż 0,05), ale nie uzyskaliśmy istotnego wyniku w teście post hoc. Przyczyny mogą być różne, np. w przypadku wspomnianego mleka p = 0,043 jest dość bliskie granicznej wartości 0,05 i w testach post hoc, w których też patrzymy na osobno policzone p, to p już przekroczyło granicę 0,05.*

Figure 3. Level of Se depending on consumption of food products (for products which differentiated Se in significant way)

**Relationship between food products and level of Zn**

Level of Zn was significantly dependent on frequency of consumption of milk and milk products (p = 0.023), offal (p = 0.045), cold cuts (p = 0.022), fresh vegetables (p = 0.004) and coffee (p = 0.042). Patients not consuming milk or milk products were characterized with higher Zn compared to patients consuming those products often (0.87 ± 0.10 mg/L vs 0.74 ± 0.11 mg/L), which was confirmed in post-hoc evaluation. In case of offal and cold cuts post hoc confirmed that level of Zn was significantly higher in case of patients consuming the products often compared to patients consuming them seldom (0.90 ± 0.16 mg/L vs 0.76 ± 0.11 mg/L and 0.80 ± 0.13 mg/L vs 0.73 ± 0.10 mg/L, respectively). Seldom consumption of fresh vegetables resulted in higher Zn, compared to often consumption (0.82 ± 0.13 mg/L vs 0.74 ± 0.09 mg/L), based on post hoc test. Growing coffee consumption was associated with higher level of Zn, however outcome of post-hoc evaluation was not significant, table 8. Significant relationships were visualized on figure 4.

Table 8. Dependance of Zn level (mg/L) on consumption of selected food products

| **Variable** | **Never** | **Seldom** | **Often** | **p** |
| --- | --- | --- | --- | --- |
| White bread | 0.79 (0.69;0.89) | 0.76 (0.73;0.81) | 0.77 (0.68;0.82) | 0.739^3^ |
| Wholemeal bread | 0.78 ± 0.11 | 0.77 ± 0.12 | 0.76 ± 0.12 | 0.969 |
| Pastries | 0.70 ± 0.10 | 0.77 ± 0.12 | 0.77 ± 0.11 | 0.388 |
| Flour products | 0.80 ± 0.12 | 0.74 ± 0.10 | 0.79 ± 0.13 | 0.075 |
| Groats/rice/cereal | 0.74 ± 0.09 | 0.78 ± 0.13 | 0.76 ± 0.11 | 0.580 |
| Milk/milk beverages | 0.87 ± 0.10^a^ | 0.78 ± 0.12 | 0.74 ± 0.11^a^ | **0.023** |
| White cheese | 0.81 ± 0.13 | 0.79 ± 0.11 | 0.74 ± 0.12 | 0.142 |
| Cheese/processed cheese | 0.76 ± 0.11 | 0.77 ± 0.12 | 0.76 ± 0.12 | 0.814 |
| Meet | - | 0.74 (0.69;0.84) | 0.77 (0.70;0.82) | 0.669^2^ |
| Poultry | - | 0.77 (0.66;0.81) | 0.76 (0.70;0.83) | 0.477^2^ |
| Offal | 0.76 ± 0.11 | 0.76 ± 0.11^b^ | 0.90 ± 0.16^b^ | **0.045** |
| Sausages | 0.72 ± 0.11 | 0.76 ± 0.11 | 0.78 ± 0.12 | 0.295 |
| Premium cold cuts | 0.73 ± 0.12 | 0.78 ± 0.11 | 0.77 ± 0.12 | 0.571 |
| Cold cuts | 0.74 ± 0.09 | 0.73 ± 0.10^c^ | 0.80 ± 0.13^c^ | **0.022** |
| Bacon/lard | 0.76 ± 0.12 | 0.77 ± 0.12 | 0.78 ± 0.12 | 0.900 |
| Meat canned food | 0.75 ± 0.11 | 0.76 ± 0.11 | 0.90 ± 0.14 | 0.054 |
| Fish canned food | 0.75 ± 0.13 | 0.78 ± 0.11 | 0.74 ± 0.12 | 0.501 |
| Fresh fish | 0.78 ± 0.19 | 0.78 ± 0.12 | 0.75 ± 0.10 | 0.426 |
| Eggs | 0.69 ± 0.09 | 0.79 ± 0.14 | 0.76 ± 0.10 | 0.161 |
| Butter | 0.77 ± 0.16 | 0.75 ± 0.12 | 0.77 ± 0.11 | 0.782 |
| Margarine | 0.75 ± 0.10 | 0.78 ± 0.12 | 0.74 ± 0.14 | 0.431 |
| Oils | 0.82 ± 0.08 | 0.79 ± 0.12 | 0.75 ± 0.12 | 0.131 |
| Potatos | - | 0.71 (0.67;0.77) | 0.77 (0.69;0.83) | 0.235^2^ |
| Boiled vegetables | 0.73 ± 0.07 | 0.76 ± 0.12 | 0.77 ± 0.12 | 0.870 |
| Fresh vegetables | 0.82 ± 0.12 | 0.82 ± 0.13^d^ | 0.74 ± 0.09^d^ | **0.004** |
| Fruit | 0.91 (0.85;0.96) | 0.81 (0.79;0.82) | 0.75 (0.68;0.82) | 0.128^3^ |
| Sugar (for beverages) | 0.77 ± 0.11 | 0.76 ± 0.13 | 0.77 ± 0.11 | 0.986 |
| Jams | 0.77 ± 0.10 | 0.77 ± 0.13 | 0.75 ± 0.08 | 0.735 |
| Honey | 0.80 ± 0.10 | 0.79 ± 0.14 | 0.74 ± 0.10 | 0.146 |
| Sweet beverages | 0.77 ± 0.10 | 0.77 ± 0.13 | 0.72 ± 0.10 | 0.738 |
| Legumes products | 0.79 ± 0.13 | 0.77 ± 0.12 | 0.74 ± 0.10 | 0.570 |
| Beer | 0.77 ± 0.13 | 0.76 ± 0.11 | 0.74 ± 0.09 | 0.866 |
| Wine | 0.78 (0.68;0.85) | 0.76 (0.70;0.81) | - | 0.823^2^ |
| Vodka | 0.77 (0.68;0.82) | 0.75 (0.70;0.82) | - | 0.993^2^ |
| Coffee | 0.69 ± 0.14 | 0.72 ± 0.10 | 0.78 ± 0.11 | **0.042** |
| Tea | 0.82 ± 0.05 | 0.76 ± 0.08 | 0.77 ± 0.12 | 0.617 |
| Vitamins | - | 0.77 ± 0.11 | 0.77 ± 0.12 | 0.992^1^ |

**Notes:** SD – standard deviation, Q1 – first quartile, Q3 – third quartile. Data presented as mean ± standard deviation or median (Q1;Q3), depending on normality of distribution. Comparisons performed with t-Student independent test^1^, Mann-Whitney U test^2^, Anova analysis or Kruskal-Wallis test^3^. Post hoc evaluation run with Tukey test. a-d – adjusted p value produced with Tukey test (a: p = 0.021, b: p = 0.034, c: p = 0.020, d: p = 0.004).

Figure 4. Level of Zn depending on consumption of food products (for products which differentiated Zn significantly)

**Relationship between food products and level of Cu**

Level of Cu was significantly dependent on frequency of consumption of eggs (p = 0.041), fruit (p = 0.046) and sweet beverages (p = 0.026). Level of Cu grew with higher consumption of eggs, however significant outcome was not accomplished in post hoc evaluation. Seldom consumption of fruit was associated with lower level of Cu compared to often consumption (median = 0.82 mg/L vs median = 1.11 mg/L), based on post hoc evaluation. Level of Cu in case of no consumption of sweet beverages was higher than in case of seldom consumption (1.24 ± 0.33 mg/L vs 1.03 ± 0.36 mg/L), which was confirmed in post hoc test, table 9. Significant relationships between Cu and food products were visualized on figure 5.

Table 9. Dependance of Cu level (mg/L) on consumption of selected food products

| **Variable** | **Never** | **Seldom** | **Often** | **p** |
| --- | --- | --- | --- | --- |
| White bread | 1.19 ± 0.45 | 1.02 ± 0.29 | 1.09 ± 0.35 | 0.537 |
| Wholemeal bread | 1.18 ± 0.63 | 1.03 ± 0.26 | 1.14 ± 0.37 | 0.313^4^ |
| Pastries | 1.10 ± 0.35 | 1.07 ± 0.29 | 1.12 ± 0.41 | 0.834 |
| Flour products | 0.85 ± 0.26 | 1.14 ± 0.36 | 1.11 ± 0.37 | 0.081 |
| Groats/rice/cereal | 1.15 (1.09;1.45) | 1.05 (0.89;1.28) | 1.12 (0.81;1.33) | 0.378^5^ |
| Milk/milk beverages | 1.14 ± 0.34 | 1.04 ± 0.39 | 1.13 ± 0.34 | 0.580 |
| White cheese | 0.92 ± 0.35 | 1.09 ± 0.37 | 1.14 ± 0.35 | 0.300 |
| Cheese/processed cheese | 1.15 ± 0.37 | 1.09 ± 0.36 | 1.10 ± 0.37 | 0.923 |
| Meet | 1.00 ± 0.43 | 1.08 ± 0.47 | 1.11 ± 0.34 | 0.806 |
| Poultry | - | 1.09 ± 0.32 | 1.10 ± 0.39 | 0.894^1^ |
| Offal | 1.20 ± 0.40 | 1.06 ± 0.33 | 0.90 ± 0.25 | 0.114 |
| Sausages | 1.05 ± 0.41 | 1.09 ± 0.35 | 1.14 ± 0.35 | 0.756 |
| Premium cold cuts | 1.11 ± 0.32 | 1.08 ± 0.43 | 1.11 ± 0.32 | 0.923 |
| Cold cuts | 1.27 ± 0.38 | 1.07 ± 0.36 | 1.07 ± 0.35 | 0.243 |
| Bacon/lard | 1.12 ± 0.37 | 1.14 ± 0.35 | 0.91 ± 0.33 | 0.106 |
| Meat canned food | 1.18 ± 0.39 | 1.06 ± 0.35 | 1.04 ± 0.21 | 0.374 |
| Fish canned food | 1.11 ± 0.43 | 1.07 ± 0.34 | 1.16 ± 0.34 | 0.657 |
| Fresh fish | 1.38 ± 0.42 | 1.08 ± 0.38 | 1.08 ± 0.33 | 0.205 |
| Eggs | 0.95 ± 0.45 | 0.98 ± 0.37 | 1.18 ± 0.32 | **0.041** |
| Butter | 1.13 ± 0.31 | 1.16 ± 0.37 | 1.08 ± 0.36 | 0.667 |
| Margarine | 1.13 ± 0.37 | 1.03 ± 0.36 | 1.21 ± 0.31 | 0.215 |
| Oils | 1.21 ± 0.28 | 1.19 ± 0.36 | 1.05 ± 0.37 | 0.240 |
| Potatos | - | 1.13 (0.87;1.64) | 1.07 (0.89;1.28) | 0.282^3^ |
| Boiled vegetables | 1.12 ± 0.15 | 1.02 ± 0.36 | 1.14 ± 0.36 | 0.370 |
| Fresh vegetables | 1.51 ± 0.40 | 1.10 ± 0.43 | 1.07 ± 0.30 | 0.053 |
| Fruit | 1.10 (1.06;1.13) | 0.82 (0.54;0.93)^a^ | 1.11 (0.91;1.34)^a^ | **0.046^5^** |
| Sugar (for beverages) | 1.15 ± 0.38 | 1.01 ± 0.34 | 1.12 ± 0.33 | 0.350 |
| Jams | 1.29 ± 0.35 | 1.04 ± 0.36 | 1.12 ± 0.34 | 0.058 |
| Honey | 1.28 (1.07;1.33) | 0.96 (0.61;1.32) | 1.09 (0.93;1.26) | 0.129^5^ |
| Sweet beverages | 1.24 ± 0.33^b^ | 1.03 ± 0.36^b^ | 0.97 ± 0.26 | **0.026** |
| Legumes products | 1.28 ± 0.24 | 1.05 ± 0.34 | 1.28 ± 0.47 | 0.051 |
| Beer | 1.15 ± 0.35 | 1.06 ± 0.38 | 0.92 ± 0.22 | 0.264 |
| Wine | 1.15 (0.94;1.33) | 1.02 (0.83;1.25) | - | 0.179 |
| Vodka | 1.14 (0.95;1.32) | 1.02 (0.81;1.25) | - | 0.138^3^ |
| Coffee | 1.10 ± 0.30 | 0.95 ± 0.39 | 1.12 ± 0.36 | 0.516^3^ |
| Tea | 1.06 ± 0.35 | 1.03 ± 0.37 | 1.12 ± 0.36 | 0.750 |
| Vitamins | - | 1.04 ± 0.37 | 1.12 ± 0.34 | 0.345^1^ |

**Notes:** SD – standard deviation, Q1 – first quartile, Q3 – third quartile. Data presented as mean ± standard deviation or median (Q1;Q3), depending on normality of distribution. Comparisons performed with t-Student independent test^1^, t-Welch independent test^2^, Mann-Whitney U test^3^, Anova or Anova Welsh analysis^4^ and Kruskal-Wallis test^5^. Post hoc evaluation run with Tukey test or Dunn test with Bonferroni adjustment. a-b – adjusted p value produced with Tukey test or Dunn test (a: p = 0.039, b: p = 0.027).

Figure 5. Level of Cu depending on consumption of food products (for products which differentiated Cu significantly)

**Relationship between food products and level of Cu/Zn**

Level of Cu/Zn was significantly dependent on frequency of consumption of flour products (p = 0.049), offal (p = 0.036), eggs (p = 0.030), fruit (p = 0.018), jams (p = 0.047) and legumes products (p = 0.042). Seldom consumption of flour products was associated with higher Cu/Zn compared to no consumption (1.60 ± 0.52 mmol vs 1.11 ± 0.35 mmol), based on post hoc test. Cu/Zn lowered with rising consumption of offal, however significant outcome of post hoc test was not accomplished. Often consumption of eggs was associated with higher Cu/Zn, compared to seldom consumption (median = 1.60 mmol vs median = 1.22 mmol), based on post hoc test. Often consumption of fruit was associated with higher Cu/Zn compared to seldom consumption (median = 1.52 mmol vs median = 0.82 mmol), based on post hoc test. No consumption of jam was associated with higher Cu/Zn compared to seldom consumption (median = 1.74 mmol vs median = 1.32 mmol), based on post hoc test. In case of legumes products Cu/Zn was higher among patients with no consumption compared to patients with seldom consumption, while patients with often consumption had the highest Cu/Zn, however significant outcome of post hoc test for legumes products was not accomplished, table 10. Significant relationships between Cu/Zn and food products were presented as figure 6.

Table 10. Dependance of Cu/Zn level (mmol) on consumption of selected food products

| **Variable** | **Never** | **Seldom** | **Often** | **p** |
| --- | --- | --- | --- | --- |
| White bread | 1.66 (1.02;1.85) | 1.43 (1.11;1.78) | 1.45 (1.20;1.76) | 0.802^3^ |
| Wholemeal bread | 1.56 ± 0.83 | 1.43 ± 0.47 | 1.56 ± 0.54 | 0.550 |
| Pastries | 1.64 ± 0.58 | 1.47 ± 0.49 | 1.51 ± 0.58 | 0.805 |
| Flour products | 1.11 ± 0.35^a^ | 1.60 ± 0.52^a^ | 1.49 ± 0.56 | **0.040** |
| Groats/rice/cereal | 1.92 ± 0.72 | 1.46 ± 0.48 | 1.48 ± 0.55 | 0.143 |
| Milk/milk beverages | 1.36 ± 0.44 | 1.41 ± 0.57 | 1.58 ± 0.53 | 0.323 |
| White cheese | 1.18 ± 0.45 | 1.42 ± 0.43 | 1.64 ± 0.60 | 0.051 |
| Cheese/processed cheese | 1.52 (1.52;1.82) | 1.43 (1.10;1.81) | 1.45 (1.18;1.78) | 0.815^3^ |
| Meet | 1.54 ± 0.75 | 1.45 ± 0.47 | 1.51 ± 0.54 | 0.935 |
| Poultry | - | 1.42 (1.20;1.77) | 1.47 (1.04;1.81) | 0.873^2^ |
| Offal | 1.74 (1.41;1.90) | 1.38 (1.13;1.71) | 1.06 (0.91;1.21) | **0.036** |
| Sausages | 1.50 ± 0.55 | 1.51 ± 0.57 | 1.53 ± 0.50 | 0.975 |
| Premium cold cuts | 1.58 ± 0.48 | 1.46 ± 0.62 | 1.52 ± 0.50 | 0.823 |
| Cold cuts | 1.79 (1.40;2.08) | 1.38 (1.20;1.75) | 1.46 (1.03;1.78) | 0.115^3^ |
| Bacon/lard | 1.54 ± 0.48 | 1.57 ± 0.56 | 1.24 ± 0.48 | 0.129 |
| Meat canned food | 1.62 ± 0.54 | 1.47 ± 0.54 | 1.22 ± 0.30 | 0.271 |
| Fish canned food | 1.53 ± 0.64 | 1.43 ± 0.44 | 1.66 ± 0.64 | 0.247 |
| Fresh fish | 1.87 ± 0.58 | 1.47 ± 0.60 | 1.49 ± 0.47 | 0.292 |
| Eggs | 1.11 (0.98;1.96) | 1.22 (0.84;1.67)^b^ | 1.60 (1.31;1.82)^b^ | **0.030^3^** |
| Butter | 1.54 ± 0.55 | 1.62 ± 0.54 | 1.46 ± 0.54 | 0.558 |
| Margarine | 1.67 (1.22;1.81) | 1.31 (1.10;1.65) | 1.68 (1.31;1.95) | 0.065^3^ |
| Oils | 1.54 ± 0.42 | 1.54 ± 0.39 | 1.49 ± 0.60 | 0.907 |
| Potatos | - | 1.73 (1.30;2.03) | 1.41 (1.11;1.76) | 0.151^2^ |
| Boiled vegetables | 1.60 ± 0.34 | 1.37 ± 0.43 | 1.56 ± 0.58 | 0.285 |
| Fresh vegetables | 1.97 (1.70;2.17) | 1.31 (0.99;1.73) | 1.47 (1.20;1.79) | 0.096^3^ |
| Fruit | 1.28 (1.15;1.40) | 0.82 (0.74;1.22)^c^ | 1.52 (1.21;1.82)^c^ | **0.018^3^** |
| Sugar (for beverages) | 1.55 ± 0.52 | 1.41 ± 0.59 | 1.53 ± 0.53 | 0.621 |
| Jams | 1.74 (1.46;1.93)^d^ | 1.32 (1.02;1.75)^d^ | 1.65 (1.33;1.76) | **0.047^3^** |
| Honey | 1.52 (1.39;1.80) | 1.18 (0.84;1.80) | 1.56 (1.28;1.79) | 0.118^3^ |
| Sweet beverages | 1.68 ± 0.47 | 1.41 ± 0.56 | 1.42 ± 0.50 | 0.099 |
| Legumes products | 1.73 (1.37;1.98) | 1.39 (1.10;1.73) | 1.85 (1.47;1.98) | **0.042^3^** |
| Beer | 1.57 ± 0.54 | 1.46 ± 0.57 | 1.27 ± 0.21 | 0.370 |
| Wine | 1.66 (1.13;1.92) | 1.39 (1.16;1.70) | - | 0.194^2^ |
| Vodka | 1.64 (1.20;1.82) | 1.39 (1.10;1.69) | - | 0.162^2^ |
| Coffee | 1.67 ± 0.57 | 1.35 ± 0.51 | 1.50 ± 0.54 | 0.490 |
| Tea | 1.33 ± 0.39 | 1.38 ± 0.43 | 1.54 ± 0.56 | 0.539 |
| Vitamins | - | 1.43 ± 0.60 | 1.53 ± 0.49 | 0.431^1^ |

**Notes:** SD – standard deviation, Q1 – first quartile, Q3 – third quartile. Data presented as mean ± standard deviation or median (Q1;Q3), depending on normality of distribution. Comparisons performed with t-Student independent test^1^, Mann-Whitney U test^2^, Anova or Kruskal-Wallis test^3^. Post hoc evaluation run with Tukey test or Dunn test with Bonferroni adjustment, as appropriate. a-d – adjusted p value produced with Tukey test or Dunn test (a: p = 0.032, b: p = 0.026, c: p = 0.017, d: p = 0.049).

Figure 6. Level of Cu/Zn depending on consumption of food products (for products which differentiated Cu/Zn significantly)

**Relationship between food products and level of TAS**

Level of TAS was not dependent on consumption of analysed food products (p > 0.05), table 11.

Table 11. Dependance of TAS level (mmol/L) on consumption of selected food products

| **Variable** | **Never** | **Seldom** | **Often** | **p** |
| --- | --- | --- | --- | --- |
| White bread | 1.56 (1.42;1.65) | 1.56 (1.48;1.60) | 1.51 (1.35;1.63) | 0.946 |
| Wholemeal bread | 1.58 (1.30;1.62) | 1.55 (1.43;1.63) | 1.53 (1.42;1.64) | 0.977 |
| Pastries | 1.50 (1.41;1.59) | 1.54 (1.38;1.62) | 1.56 (1.43;1.65) | 0.784 |
| Flour products | 1.56 (1.43;1.65) | 1.47 (1.42;1.58) | 1.59 (1.43;1.65) | 0.155 |
| Groats/rice/cereal | 1.60 (1.52;1.67) | 1.47 (1.41;1.63) | 1.56 (1.42;1.62) | 0.402 |
| Milk/milk beverages | 1.61 (1.55;1.65) | 1.56 (1.41;1.64) | 1.47 (1.42;1.59) | 0.187 |
| White cheese | 1.59 (1.50;1.74) | 1.55 (1.30;1.65) | 1.51 (1.42;1.61) | 0.403 |
| Cheese/processed cheese | 1.65 (1.54;1.66) | 1.47 (1.39;1.61) | 1.57 (1.43;1.64) | 0.093 |
| Meet | 1.56 (1.50;1.63) | 1.52 (1.36;1.60) | 1.54 (1.42;1.64) | 0.870 |
| Poultry | - | 1.56 (1.41;1.63) | 1.50 (1.42;1.62) | 0.678^1^ |
| Offal | 1.53 (1.43;1.63) | 1.54 (1.35;1.62) | - | 0.900^1^ |
| Sausages | 1.59 (1.41;1.65) | 1.51 (1.35;1.62) | 1.55 (1.43;1.59) | 0.916 |
| Premium cold cuts | 1.55 (1.41;1.62) | 1.55 (1.43;1.63) | 1.54 (1.41;1.64) | 0.957 |
| Cold cuts | 1.56 (1.43;1.61) | 1.47 (1.42;1.60) | 1.57 (1.43;1.65) | 0.380 |
| Bacon/lard | 1.55 (1.42;1.64) | 1.55 (1.42;1.64) | 1.47 (1.30;1.57) | 0.598 |
| Meat canned food | 1.58 (1.43;1.65) | 1.49 (1.42;1.62) | 1.44 (1.23;1.69) | 0.709 |
| Fish canned food | 1.58 (1.46;1.65) | 1.55 (1.38;1.63) | 1.44 (1.42;1.62) | 0.574 |
| Fresh fish | 1.50 (1.50;1.58) | 1.58 (1.47;1.65) | 1.44 (1.35;1.60) | 0.188 |
| Eggs | 1.47 (1.47;1.50) | 1.56 (1.42;1.70) | 1.53 (1.42;1.63) | 0.465 |
| Butter | 1.54 (1.49;1.64) | 1.45 (1.43;1.62) | 1.55 (1.41;1.64) | 0.658 |
| Margarine | 1.48 (1.42;1.61) | 1.58 (1.42;1.66) | 1.47 (1.32;1.58) | 0.160 |
| Oils | 1.46 (1.33;1.56) | 1.51 (1.43;1.58) | 1.55 (1.42;1.65) | 0.363 |
| Potatos | - | 1.46 (1.38;1.58) | 1.55 (1.42;1.64) | 0.215^1^ |
| Boiled vegetables | 1.31 (1.22;1.46) | 1.48 (1.41;1.62) | 1.56 (1.43;1.65) | 0.295 |
| Fresh vegetables | 1.62 (1.54;1.66) | 1.56 (1.43;1.64) | 1.51 (1.37;1.61) | 0.536 |
| Fruit | 1.90 (1.79;2.00) | 1.64 (1.35;1.67) | 1.52 (1.42;1.61) | 0.090 |
| Sugar (for beverages) | 1.56 (1.42;1.64) | 1.47 (1.39;1.67) | 1.56 (1.43;1.62) | 0.965 |
| Jams | 1.58 (1.43;1.66) | 1.51 (1.42;1.64) | 1.54 (1.28;1.61) | 0.619 |
| Honey | 1.55 (1.48;1.65) | 1.45 (1.36;1.64) | 1.55 (1.43;1.63) | 0.358 |
| Sweet beverages | 1.55 (1.44;1.63) | 1.55 (1.35;1.63) | 1.54 (1.51;1.70) | 0.740 |
| Legumes products | 1.47 (1.39;1.66) | 1.53 (1.41;1.63) | 1.58 (1.53;1.59) | 0.620 |
| Beer | 1.55 (1.42;1.63) | 1.56 (1.41;1.64) | 1.45 (1.43;1.53) | 0.824 |
| Wine | 1.55 (1.44;1.64) | 1.49 (1.37;1.63) | - | 0.692^1^ |
| Vodka | 1.54 (1.43;1.61) | 1.55 (1.41;1.65) | - | 0.619^1^ |
| Coffee | 1.49 (1.37;1.58) | 1.59 (1.52;1.68) | 1.55 (1.42;1.64) | 0.538 |
| Tea | 1.46 ± 0.64 | 1.62 ± 0.14 | 1.49 ± 0.22 | 0.143^2^ |
| Vitamins | - | 1.56 (1.46;1.61) | 1.51 (1.34;1.64) | 0.392^1^ |

**Notes:** SD – standard deviation, Q1 – first quartile, Q3 – third quartile. Data presented as mean ± standard deviation or median (Q1;Q3), depending on normality of distribution. Comparisons performed with Mann-Whitney U test^1^, Anova Welch^2^ analysis or Kruskal-Wallis test.

**Relationship between food products and eye (left/right)**

Significance of the difference in consumption of selected food products between left eye and right eye was verified. No relationship was found significant (p > 0.05), table 12.

Table 12. Correlation of eye (left/right) and consumption of selected food products

| **Variable** | **Left eye** | | | **Right eye** | | | **p** |
| --- | --- | --- | --- | --- | --- | --- | --- |
|  | **Never** | **Seldom** | **Often** | **Never** | **Seldom** | **Often** |  |
| White bread | 7 (16.3) | 5 (11.6) | 31 (72.1) | 5 (12.5) | 5 (12.5) | 30 (75.0) | 0.886^1^ |
| Wholemeal bread | 4 (9.1) | 19 (43.2) | 21 (47.7) | 3 (7.5) | 16 (40.0) | 21 (52.5) | 0.947 |
| Pastries | 3 (6.8) | 17 (38.6) | 24 (54.5) | 2 (5.0) | 18 (45.0) | 20 (50.0) | 0.831 |
| Flour products | 6 (13.6) | 21 (47.7) | 17 (38.6) | 3 (7.5) | 21 (52.5) | 16 (40.0) | 0.656^1^ |
| Groats/rice/cereal | 5 (11.4) | 19 (43.2) | 20 (45.5) | 1 (2.5) | 19 (47.5) | 20 (50.0) | 0.323 |
| Milk/milk beverages | 2 (4.5) | 16 (36.4) | 26 (59.1) | 5 (12.5) | 12 (30.0) | 23 (57.5) | 0.422 |
| White cheese | 5 (11.4) | 18 (40.9) | 21 (47.7) | 2 (5.0) | 18 (45.0) | 20 (50.0) | 0.642 |
| Cheese/processed cheese | 5 (11.4) | 22 (50.0) | 17 (38.6) | 0 (0.0) | 22 (55.0) | 18 (45.0) | 0.104 |
| Meet | 4 (9.1) | 3 (6.8) | 37 (84.1) | 1 (2.5) | 7 (17.5) | 32 (80.0) | 0.171 |
| Poultry | 0 (0.0) | 24 (54.5) | 20 (45.5) | 1 (2.5) | 16 (40.0) | 23 (57.5) | 0.229 |
| Offal | 14 (31.8) | 28 (63.6) | 2 (4.5) | 14 (35.0) | 24 (60.0) | 2 (5.0) | 0.930 |
| Sausages | 9 (21.4) | 22 (52.4) | 11 (26.2) | 5 (12.5) | 19 (47.5) | 16 (40.0) | 0.326^1^ |
| Premium cold cuts | 5 (11.4) | 15 (34.1) | 24 (54.5) | 3 (7.5) | 14 (35.0) | 23 (57.5) | 0.897 |
| Cold cuts | 6 (13.6) | 15 (34.1) | 23 (52.3) | 5 (12.5) | 17 (42.5) | 18 (45.0) | 0.727^1^ |
| Bacon/lard | 9 (20.5) | 27 (61.4) | 8 (18.2) | 12 (30.0) | 22 (55.0) | 6 (15.0) | 0.596^1^ |
| Meat canned food | 12 (27.3) | 29 (65.9) | 3 (6.8) | 14 (35.0) | 25 (62.5) | 1 (2.5) | 0.647 |
| Fish canned food | 6 (13.6) | 27 (61.4) | 11 (25.0) | 10 (25.0) | 20 (50.0) | 10 (25.0) | 0.386^1^ |
| Fresh fish | 3 (6.8) | 20 (45.5) | 21 (47.7) | 2 (5.0) | 16 (40.0) | 22 (55.0) | 0.830 |
| Eggs | 4 (9.1) | 14 (31.8) | 26 (59.1) | 1 (2.5) | 15 (37.5) | 24 (60.0) | 0.518 |
| Butter | 5 (11.4) | 9 (20.5) | 30 (68.2) | 2 (5.0) | 9 (22.5) | 29 (72.5) | 0.595 |
| Margarine | 16 (36.4) | 21 (47.7) | 7 (15.9) | 17 (42.5) | 16 (40.0) | 7 (17.5) | 0.772^1^ |
| Oils | 4 (9.1) | 10 (22.7) | 30 (68.2) | 6 (15.0) | 7 (17.5) | 27 (67.5) | 0.638^1^ |
| Potatos | 0 (0.0) | 5 (11.6) | 38 (88.4) | 1 (2.5) | 7 (17.5) | 32 (80.0) | 0.440 |
| Boiled vegetables | 3 (6.8) | 11 (25.0) | 30 (68.2) | 0 (0.0) | 15 (37.5) | 25 (62.5) | 0.154 |
| Fresh vegetables | 1 (2.3) | 15 (34.1) | 28 (63.6) | 3 (7.5) | 11 (27.5) | 26 (65.0) | 0.485 |
| Fruit | 1 (2.3) | 2 (4.5) | 41 (93.2) | 1 (2.5) | 5 (12.5) | 34 (85.0) | 0.467 |
| Sugar (for beverages) | 14 (31.8) | 14 (31.8) | 16 (36.4) | 22 (55.0) | 10 (25.0) | 8 (20.0) | 0.085^1^ |
| Jams | 5 (11.4) | 27 (61.4) | 12 (27.3) | 10 (25.0) | 24 (60.0) | 6 (15.0) | 0.160^1^ |
| Honey | 7 (15.9) | 16 (36.4) | 21 (47.7) | 6 (15.0) | 10 (25.0) | 24 (60.0) | 0.478^1^ |
| Sweet beverages | 13 (29.5) | 28 (63.6) | 3 (6.8) | 17 (43.6) | 21 (53.8) | 1 (2.6) | 0.307 |
| Legumes products | 4 (9.1) | 35 (79.5) | 5 (11.4) | 4 (10.0) | 31 (77.5) | 5 (12.5) | > 0.999 |
| Beer | 21 (47.7) | 19 (43.2) | 4 (9.1) | 23 (57.5) | 15 (37.5) | 2 (5.0) | 0.629 |
| Wine | 22 (50.0) | 22 (50.0) | 0 (0.0) | 19 (48.7) | 20 (51.3) | 0 (0.0) | > 0.999^1^ |
| Vodka | 22 (50.0) | 22 (50.0) | 0 (0.0) | 23 (57.5) | 17 (42.5) | 0 (0.0) | 0.639^1^ |
| Coffee | 6 (13.6) | 4 (9.1) | 34 (77.3) | 4 (10.3) | 2 (5.1) | 33 (84.6) | 0.770 |
| Tea | 3 (6.8) | 5 (11.4) | 36 (81.8) | 2 (5.1) | 4 (10.3) | 33 (84.6) | > 0.999 |
| Vitamins | 0 (0.0) | 12 (26.7) | 33 (73.3) | 0 (0.0) | 16 (37.2) | 27 (62.8) | 0.405^1^ |

**Notes:** Data presented as n (% of left eye or right eye group). Comparisons performed with Pearson chi-square test^1^ or Fisher exact test.

*W tabeli 12 mamy zależność częstość konsumpcji od oka (lewe/prawe), czyli inaczej: sprawdzamy, czy struktura częstości konsumpcji różniła się w przypadku oka lewego i prawego. Częstości konsumpcji są pokazane jako liczba obserwacji i %, przy czym trzy pozycje dla oka lewego sumują się do 100% i tak samo trzy pozycje dla oka prawego sumują się do 100%. p oznacza poziom istotności różnicy między tymi strukturami. (Podobnie mamy w tabeli 18, przy czym tam zamiast oka lewe/prawe mamy płeć).*

**Relationship between food products and IOP**

Level of IOP varied depending on consumption of milk and milk products (p = 0.43). Seldom consumption was associated with higher IOP compared to often consumption (median = 22.95 mmHg vs median = 18.70 mmHg), table 13. Level of IOP for patients with different frequency of milk and milk products consumption was presented as figure 7.

Table 13. Dependance of IOP (mmHg) on consumption of selected food products

| **Variable** | **Never** | **Seldom** | **Often** | **p** |
| --- | --- | --- | --- | --- |
| White bread | 22.15 (18.00;28.30) | 21.95 (17.27;25.50) | 19.70 (15.80;25.00) | 0.543 |
| Wholemeal bread | 23.00 (19.65;26.00) | 19.70 (15.30;23.65) | 19.55 (16.30;26.38) | 0.464 |
| Pastries | 22.30 (21.30;31.00) | 19.70 (15.50;24.30) | 19.65 (15.78;25.08) | 0.206 |
| Flour products | 25.30 (19.40;30.00) | 19.00 (15.07;23.82) | 20.90 (16.30;24.00) | 0.189 |
| Groats/rice/cereal | 18.20 (15.20;21.12) | 20.50 (16.18;25.23) | 19.00 (16.15;25.40) | 0.771 |
| Milk/milk beverages | 20.30 (17.00;21.85) | 22.95 (19.22;29.25)^a^ | 18.70 (15.70;22.30)^a^ | **0.043** |
| White cheese | 23.00 (20.05;33.85) | 20.45 (16.23;25.40) | 18.70 (15.00;23.00) | 0.066 |
| Cheese/processed cheese | - | 19.70 (15.00;23.48) | 19.00 (16.15;25.50) | 0.372^1^ |
| Meet | 21.30 (18.70;22.30) | 21.55 (18.20;24.80) | 19.40 (15.80;25.30) | 0.769 |
| Poultry | - | 20.30 (17.05;25.40) | 19.00 (15.50;23.50) | 0.327^1^ |
| Offal | 20.35 (16.30;25.48) | 19.70 (15.30;24.25) | - | 0.335^1^ |
| Sausages | 20.35 (16.03;22.82) | 20.20 (16.30;25.70) | 20.30 (15.65;25.65) | 0.901 |
| Premium cold cuts | 19.50 (16.18;23.05) | 20.30 (16.00;25.30) | 19.00 (15.85;24.30) | 0.836 |
| Cold cuts | 19.00 (16.70;23.30) | 19.00 (14.93;23.08) | 21.00 (16.00;26.70) | 0.270 |
| Bacon/lard | 20.70 (16.00;25.00) | 19.00 (15.30;24.00) | 20.75 (16.98;28.82) | 0.308 |
| Meat canned food | 18.20 (15.20;22.82) | 20.30 (16.40;25.60) | - | 0.183^1^ |
| Fish canned food | 19.85 (14.93;23.00) | 20.30 (16.70;25.30) | 17.30 (15.30;25.30) | 0.572 |
| Fresh fish | 21.30 (20.70;25.30) | 19.55 (15.75;25.08) | 19.70 (15.90;24.65) | 0.680 |
| Eggs | 21.30 (19.40;23.00) | 20.20 (16.00;26.50) | 19.35 (15.43;23.82) | 0.817 |
| Butter | 19.40 (17.65;21.00) | 20.25 (15.07;21.98) | 19.70 (16.00;25.50) | 0.910 |
| Margarine | 19.40 (16.30;26.00) | 20.30 (16.00;25.70) | 19.35 (15.07;20.85) | 0.399 |
| Oils | 19.65 (15.32;24.75) | 20.30 (15.00;25.30) | 19.70 (16.00;25.00) | 0.994 |
| Potatos | - | 21.10 (14.27;25.40) | 20.00 (16.30;25.23) | 0.865^1^ |
| Boiled vegetables | 29.00 (21.85;32.50) | 19.55 (16.70;22.67) | 20.30 (15.75;25.30) | 0.712 |
| Fresh vegetables | 18.77 ± 4.68 | 19.46 ± 4.31 | 22.45 ± 8.29 | 0.153^2^ |
| Fruit | 32.00 (29.50;34.50) | 20.90 (17.60;22.95) | 19.40 (15.90;25.15) | 0.148 |
| Sugar (for beverages) | 20.85 (16.23;23.48) | 20.25 (16.35;26.55) | 18.20 (15.23;24.32) | 0.535 |
| Jams | 21.00 (16.05;25.30) | 19.70 (16.70;24.50) | 18.30 (15.00;24.45) | 0.712 |
| Honey | 21.00 (16.30;25.00) | 19.95 (15.43;22.98) | 18.70 (16.00;25.30) | 0.840 |
| Sweet beverages | 20.85 (16.70;25.23) | 19.00 (15.30;24.00) | - | 0.207^1^ |
| Legumes products | 19.85 (16.47;21.08) | 19.70 (16.00;25.23) | 23.00 (15.32;25.30) | 0.937 |
| Beer | 19.35 (15.95;23.80) | 19.95 (15.77;25.27) | 22.80 (19.40;25.23) | 0.632 |
| Wine | 19.70 (15.00;25.30) | 20.25 (16.08;24.98) | - | 0.848^1^ |
| Vodka | 20.30 (16.00;25.30) | 19.70 (15.85;25.15) | - | 0.760^1^ |
| Coffee | 20.00 (15.68;24.55) | 18.10 (15.25;21.47) | 20.30 (16.15;25.30) | 0.789 |
| Tea | 30.70 (23.30;37.00) | 19.00 (15.00;21.90) | 19.70 (16.00;25.30) | 0.137 |
| Vitamins | - | 19.00 (16.60;24.00) | 20.45 (15.68;25.30) | 0.826^1^ |

**Notes:** SD – standard deviation, Q1 – first quartile, Q3 – third quartile. Data presented as mean ± standard deviation or median (Q1;Q3), depending on normality of distribution. Comparisons performed with Mann-Whitney U test^1^, Anova Welch^2^ analysis or Kruskal-Wallis test. Post hoc evaluation run with Dunn test with Bonferroni adjustment. a – adjusted p value produced with Dunn test (p = 0.039).

Figure 7. Level of IOP depending on consumption of milk and milk products

**Relationship between food products and BCVA**

Level of BCVA was significantly dependent on frequency of consumption of white cheese (p = 0.008) and boiled vegetables (p = 0.048). Seldom consumption of white cheese was associated with higher BCVA compared to often consumption (median = 0.90 vs median = 0.60), based on post hoc test. Growing frequency of consumption of boiled vegetables was associated with lowered BCVA, however significant outcome of post hoc test was not accomplished, table 14.

Table 14. Dependance of BCVA level on consumption of selected food products

| **Variable** | **Never** | **Seldom** | **Often** | **p** |
| --- | --- | --- | --- | --- |
| White bread | 0.60 (0.42;1.00) | 0.85 (0.72;1.00) | 0.70 (0.40;0.90) | 0.386 |
| Wholemeal bread | 0.80 (0.60;0.95) | 0.70 (0.50;0.90) | 0.70 (0.23;1.00) | 0.811 |
| Pastries | 0.80 (0.70;0.90) | 0.80 (0.45;1.00) | 0.70 (0.20;0.90) | 0.508 |
| Flour products | 0.60 (0.40;1.00) | 0.80 (0.50;1.00) | 0.70 (0.20;0.90) | 0.439 |
| Groats/rice/cereal | 0.65 (0.52;0.85) | 0.80 (0.40;0.90) | 0.70 (0.40;1.00) | 0.999 |
| Milk/milk beverages | 0.90 (0.55;0.95) | 0.80 (0.58;1.00) | 0.60 (0.30;0.90) | 0.375 |
| White cheese | 0.90 (0.55;1.00) | 0.90 (0.67;1.00)^a^ | 0.60 (0.20;0.80)^a^ | **0.008** |
| Cheese/processed cheese | 0.80 (0.70;1.00) | 0.75 (0.40;0.92) | 0.70 (0.35;0.90) | 0.521 |
| Meet | 0.70 (0.50;0.80) | 0.65 (0.18;0.90) | 0.70 (0.40;1.00) | 0.851 |
| Poultry | - | 0.80 (0.35;1.00) | 0.70 (0.45;0.90) | 0.941^1^ |
| Offal | 0.65 (0.38;1.00) | 0.70 (0.40;0.90) | - | 0.996^1^ |
| Sausages | 0.65 (0.32;0.95) | 0.60 (0.30;0.90) | 0.80 (0.55;1.00) | 0.450 |
| Premium cold cuts | 0.55 (0.45;0.72) | 0.70 (0.20;0.90) | 0.80 (0.40;1.00) | 0.352 |
| Cold cuts | 0.70 (0.45;0.95) | 0.80 (0.27;1.00) | 0.70 (0.40;0.90) | 0.927 |
| Bacon/lard | 0.70 (0.50;0.90) | 0.70 (0.40;1.00) | 0.80 (0.52;0.98) | 0.742 |
| Meat canned food | 0.65 (0.40;0.90) | 0.70 (0.40;0.98) | - | 0.686^1^ |
| Fish canned food | 0.65 (0.40;0.90) | 0.70 (0.25;0.90) | 0.80 (0.60;1.00) | 0.389 |
| Fresh fish | 0.80 (0.70;0.90) | 0.70 (0.38;0.90) | 0.80 (0.40;1.00) | 0.704 |
| Eggs | 0.60 (0.20;0.70) | 0.90 (0.50;0.90) | 0.60 (0.40;0.98) | 0.453 |
| Butter | 0.70 (0.40;0.95) | 0.55 (0.20;0.88) | 0.70 (0.45;1.00) | 0.306 |
| Margarine | 0.80 (0.40;1.00) | 0.70 (0.30;0.90) | 0.80 (0.62;0.90) | 0.745 |
| Oils | 0.90 (0.58;0.98) | 0.90 (0.40;1.00) | 0.70 (0.40;0.90) | 0.303 |
| Potatos | - | 0.70 (0.48;1.00) | 0.70 (0.40;0.90) | 0.745^1^ |
| Boiled vegetables | 1.00 (1.00;1.00) | 0.80 (0.52;0.90) | 0.60 (0.35;0.90) | **0.048** |
| Fresh vegetables | 0.55 (0.25;0.83) | 0.80 (0.43;0.90) | 0.70 (0.32;1.00) | 0.667 |
| Fruit | 0.55 (0.32;0.78) | 0.80 (0.50;0.90) | 0.70 (0.40;0.95) | 0.983 |
| Sugar (for beverages) | 0.75 (0.27;0.92) | 0.70 (0.48;0.90) | 0.70 (0.50;1.00) | 0.880 |
| Jams | 0.80 (0.45;0.85) | 0.70 (0.35;0.90) | 0.75 (0.50;1.00) | 0.819 |
| Honey | 0.80 (0.70;1.00) | 0.70 (0.43;0.90) | 0.70 (0.30;1.00) | 0.479 |
| Sweet beverages | 0.80 (0.40;0.90) | 0.70 (0.40;0.90) | - | 0.740^1^ |
| Legumes products | 0.80 (0.25;0.92) | 0.80 (0.40;1.00) | 0.60 (0.52;0.67) | 0.512 |
| Beer | 0.80 (0.38;1.00) | 0.65 (0.40;0.90) | 0.85 (0.72;0.90) | 0.378 |
| Wine | 0.80 (0.40;1.00) | 0.70 (0.40;0.90) | - | 0.499^1^ |
| Vodka | 0.80 (0.40;1.00) | 0.70 (0.35;0.90) | - | 0.488^1^ |
| Coffee | 0.70 (0.20;0.90) | 0.55 (0.43;0.90) | 0.70 (0.40;0.90) | 0.869 |
| Tea | 0.60 ± 0.39 | 0.45 ± 0.41 | 0.67 ± 0.31 | 0.180^2^ |
| Vitamins | - | 0.70 (0.38;0.92) | 0.70 (0.40;0.90) | 0.846^1^ |

**Notes:** SD – standard deviation, Q1 – first quartile, Q3 – third quartile. Data presented as mean ± standard deviation or median (Q1;Q3), depending on normality of distribution. Comparisons performed with Mann-Whitney U test^1^, Anova^2^ or Kruskal-Wallis test. Post hoc evaluation run with Dunn test with Bonferroni adjustment. a – adjusted p value produced with Dunn test (p = 0.010).

Figure 8. Level of BCVA depending on consumption of white cheese and boiled vegetables (significantly relationships)

**Relationship between food products and RNFL**

Level of RNFL was not dependent on consumption of analysed food products (p > 0.05), table 15.

Table 15. Dependance of RNFL on consumption of selected food products

| **Variable** | **Never** | **Seldom** | **Often** | **p** |
| --- | --- | --- | --- | --- |
| White bread | 84.00 (71.50;102.00) | 76.00 (54.00;81.00) | 67.00 (52.50;90.75) | 0.139^3^ |
| Wholemeal bread | 71.14 ± 20.34 | 73.30 ± 22.81 | 71.31 ± 24.91 | 0.933 |
| Pastries | 75.40 ± 36.76 | 70.42 ± 22.06 | 73.10 ± 23.25 | 0.846 |
| Flour products | 70.78 ± 24.91 | 74.03 ± 22.74 | 70.25 ± 24.38 | 0.789 |
| Groats/rice/cereal | 61.50 ± 23.83 | 76.60 ± 24.24 | 69.68 ± 22.28 | 0.234 |
| Milk/milk beverages | 72.83 ± 20.00 | 73.12 ± 22.85 | 71.49 ± 24.53 | 0.959 |
| White cheese | 79.00 (60.00;97.00) | 73.00 (54.00;87.00) | 67.00 (51.00;92.00) | 0.675^3^ |
| Cheese/processed cheese | 66.75 ± 23.63 | 70.20 ± 23.16 | 74.94 ± 24.04 | 0.616 |
| Meet | 50.20 ± 10.85 | 79.44 ± 21.62 | 72.80 ± 23.60 | 0.068 |
| Poultry | - | 73.00 (54.00;93.00) | 67.00 (48.00;84.00) | 0.337^2^ |
| Offal | 68.19 ± 25.92 | 73.43 ± 22.36 | 86.33 ± 10.21 | 0.369 |
| Sausages | 66.57 ± 24.89 | 72.23 ± 23.55 | 72.67 ± 21.92 | 0.699 |
| Premium cold cuts | 66.88 ± 26.08 | 69.11 ± 22.65 | 75.07 ± 23.56 | 0.467 |
| Cold cuts | 70.91 ± 23.96 | 66.64 ± 23.24 | 76.30 ± 23.15 | 0.245 |
| Bacon/lard | 72.90 ± 26.33 | 72.30 ± 24.14 | 70.08 ± 15.49 | 0.944 |
| Meat canned food | 68.44 ± 21.65 | 73.44 ± 24.90 | 78.75 ± 13.60 | 0.584 |
| Fish canned food | 70.60 ± 20.22 | 71.63 ± 24.55 | 74.24 ± 24.10 | 0.883 |
| Fresh fish | 64.40 ± 17.39 | 71.68 ± 24.58 | 73.47 ± 23.35 | 0.714 |
| Eggs | 54.50 ± 11.73 | 76.04 ± 26.79 | 71.30 ± 21.51 | 0.214 |
| Butter | 61.83 ± 26.26 | 70.12 ± 23.62 | 73.77 ± 23.19 | 0.465 |
| Margarine | 70.48 ± 24.60 | 73.34 ± 24.50 | 72.77 ± 18.41 | 0.882 |
| Oils | 68.50 (56.75;71.50) | 73.00 (58.00;90.00) | 71.00 (48.00;93.75) | 0.648^3^ |
| Potatos | - | 80.00 (64.75;102.00) | 70.00 (49.00;86.00) | 0.219^2^ |
| Boiled vegetables | 48.00 (42.00;67.00) | 80.00 (57.00;96.00) | 68.00 (51.00;88.00) | 0.266^3^ |
| Fresh vegetables | 73.25 ± 25.50 | 75.68 ± 23.50 | 70.26 ± 23.51 | 0.643 |
| Fruit | 100.00 (99.00;101.00) | 76.00 (63.50;87.75) | 68.00 (48.50;88.00) | 0.170^3^ |
| Sugar (for beverages) | 67.00 (51.50;84.50) | 80.00 (43.00;91.50) | 71.00 (63.00;96.00) | 0.432^3^ |
| Jams | 69.07 ± 23.92 | 72.92 ± 23.04 | 72.41 ± 25.30 | 0.866 |
| Honey | 74.64 ± 22.37 | 75.38 ± 24.45 | 69.73 ± 23.35 | 0.598 |
| Sweet beverages | 71.07 ± 22.99 | 72.91 ± 24.55 | 76.25 ± 15.46 | 0.898 |
| Legumes products | 77.25 ± 21.65 | 72.70 ± 24.05 | 64.50 ± 21.06 | 0.483 |
| Beer | 76.31 ± 21.55 | 65.00 ± 25.14 | 79.67 ± 20.64 | 0.088 |
| Wine | 71.00 (55.50;90.50) | 70.00 (46.50;91.00) | - | 0.469^2^ |
| Vodka | 71.00 (56.50;88.00) | 68.00 (45.25;93.75) | - | 0.425^2^ |
| Coffee | 69.20 ± 23.45 | 60.83 ± 28.44 | 74.06 ± 22.96 | 0.380 |
| Tea | 81.00 ± 26.80 | 61.57 ± 23.38 | 72.92 ± 23.16 | 0.337 |
| Vitamins | - | 72.38 ± 21.69 | 71.00 ± 23.49 | 0.799^1^ |

**Notes:** SD – standard deviation, Q1 – first quartile, Q3 – third quartile. Data presented as mean ± standard deviation or median (Q1;Q3), depending on normality of distribution. Comparisons performed with t-Student independent test^1^, Mann-Whitney U test^2^, Anova analysis or Kruskal-Wallis test^3^.

**Relationship between food products and age**

Age was significantly related to frequency of consumption of white cheese (p = 0.017), meat canned food (p = 0.035) and legumes products (p = 0.006). Patients consuming white cheese often were significantly older than patients consuming it seldom (median = 74.00 years vs median = 67.00 years), based on post hoc test. Patients who did not eat canned meat were significantly older than patients consuming it both seldom and often (74.88 ± 6.59 years vs 68.63 ± 10.87 years), based on post hoc evaluation. Median age was significantly lower among patients consuming legumes products seldom, compared to the ones consuming those products often (median = 71.00 years vs median = 76.50 years), based on post hoc test, table 16. Visualization of age distribution by consumption groups, for food products differentiating age significantly, was presented in figure 9.

Table 16. Dependance of age (years) on consumption of selected food products

| **Variable** | **Never** | **Seldom** | **Often** | **p** |
| --- | --- | --- | --- | --- |
| White bread | 72.00 (67.00;74.00) | 66.50 (52.00;73.25) | 73.00 (66.00;77.00) | 0.322 |
| Wholemeal bread | 74.00 (71.00;77.00) | 73.00 (63.00;76.00) | 70.00 (66.00;75.75) | 0.611 |
| Pastries | 75.00 (67.00;80.00) | 71.50 (66.00;75.00) | 73.00 (65.25;76.00) | 0.704 |
| Flour products | 74.00 (67.00;76.00) | 72.00 (66.00;75.00) | 74.00 (63.00;77.00) | 0.601 |
| Groats/rice/cereal | 67.00 ± 19.58 | 69.05 ± 9.21 | 71.45 ± 10.85 | 0.492^2^ |
| Milk/milk beverages | 74.00 (69.50;75.00) | 70.00 (65.75;75.25) | 73.00 (65.50;76.25) | 0.787 |
| White cheese | 74.00 (67.50;75.00) | 67.00 (61.00;74.00)^a^ | 74.00 (70.50;78.50)^a^ | **0.017** |
| Cheese/processed cheese | 75.60 ± 5.55 | 69.70 ± 9.79 | 69.71 ± 12.71 | 0.508^2^ |
| Meet | 80.00 (75.00;82.00) | 69.00 (66.00;76.75) | 72.50 (63.75;75.00) | 0.230 |
| Poultry | - | 71.00 (66.00;74.00) | 74.00 (61.50;79.50) | 0.200^1^ |
| Offal | 74.00 (65.00;79.50) | 72.50 (66.00;75.00) | 73.00 (62.25;80.25) | 0.683 |
| Sausages | 72.50 (67.00;81.50) | 71.50 (66.00;75.25) | 74.00 (61.50;75.50) | 0.640 |
| Premium cold cuts | 78.00 (72.75;80.50) | 72.00 (62.00;75.00) | 71.50 (66.00;75.00) | 0.209 |
| Cold cuts | 77.00 (68.50;83.75) | 72.00 (66.00;74.00) | 72.00 (64.00;77.00) | 0.211 |
| Bacon/lard | 73.65 ± 8.13 | 70.18 ± 9.02 | 64.50 ± 17.28 | 0.130^3^ |
| Meat canned food | 74.88 ± 6.59^bc^ | 68.63 ± 10.87^b^ | 59.25 ± 20.56^c^ | **0.035^3^** |
| Fish canned food | 72.07 ± 4.80 | 69.00 ± 12.64 | 71.00 ± 9.83 | 0.580^2^ |
| Fresh fish | 78.00 (69.00;79.00) | 71.00 (62.75;76.00) | 73.00 (66.00;75.00) | 0.564 |
| Eggs | 74.00 (67.00;76.00) | 71.00 (65.75;75.00) | 73.00 (66.00;76.75) | 0.867 |
| Butter | 76.00 (74.50;79.00) | 73.00 (68.00;78.25) | 71.00 (63.25;75.00) | 0.058 |
| Margarine | 71.22 ± 8.65 | 67.59 ± 12.54 | 73.93 ± 9.93 | 0.134^2^ |
| Oils | 71.00 (66.00;74.00) | 72.00 (66.00;76.00) | 73.00 (66.00;77.00) | 0.706 |
| Potatos | - | 73.00 (69.75;77.50) | 72.00 (65.00;75.00) | 0.432^1^ |
| Boiled vegetables | 67.00 (66.50;68.00) | 66.50 (61.50;75.50) | 74.00 (68.25;76.00) | 0.132 |
| Fresh vegetables | 79.00 (73.00;82.50) | 74.00 (67.00;76.75) | 72.00 (63.25;75.00) | 0.291 |
| Fruit | 67.50 (67.25;67.75) | 66.00 (61.50;74.50) | 73.00 (66.00;76.00) | 0.573 |
| Sugar (for beverages) | 72.00 (66.00;76.50) | 73.00 (65.75;74.25) | 72.50 (62.00;76.75) | 0.840 |
| Jams | 72.93 ± 9.55 | 70.14 ± 9.45 | 67.44 ± 15.02 | 0.357^2^ |
| Honey | 69.15 ± 8.66 | 68.35 ± 13.49 | 71.34 ± 9.80 | 0.517^2^ |
| Sweet beverages | 74.00 (66.00;78.00) | 72.00 (63.00;75.00) | 74.00 (63.25;83.00) | 0.398 |
| Legumes products | 76.00 (67.00;79.00) | 71.00 (62.25;74.75)^d^ | 76.50 (75.00;78.75)^d^ | **0.006** |
| Beer | 71.35 ± 9.35 | 70.18 ± 10.82 | 60.17 ± 17.59 | 0.061^2^ |
| Wine | 72.50 (66.00;77.00) | 73.00 (62.25;75.00) | - | 0.534^1^ |
| Vodka | 72.00 (66.00;76.25) | 73.00 (61.50;75.50) | - | 0.622^1^ |
| Coffee | 70.00 (66.25;72.75) | 79.00 (75.50;84.75) | 73.00 (63.50;75.75) | 0.105 |
| Tea | 71.40 ± 3.78 | 72.44 ± 9.76 | 69.69 ± 11.49 | 0.754^2^ |
| Vitamins | - | 72.00 (64.00;74.50) | 73.00 (66.00;77.00) | 0.666^1^ |

**Notes:** SD – standard deviation, Q1 – first quartile, Q3 – third quartile. Data presented as mean ± standard deviation or median (Q1;Q3), depending on normality of distribution. Comparisons performed with Mann-Whitney U test^1^, Anova^2^ or Anova Welch^3^ analysis or Kruskal-Wallis test. Post hoc evaluation run with Tukey test or Dunn test with Bonferroni adjustment. a-d – adjusted p value produced with Tukey test or Dunn test (a: p = 0.014, b: p = 0.039, c: p = 0.017, d: p = 0.008).

Figure 9. Age within consumption groups (for products which differentiated age significantly)

**Relationship between food products and BMI**

BMI was significantly related to frequency of consumption of margarine (p = 0.046) and oils (p = 0.015). Patients never consuming margarine had significantly higher BMI than patients consuming it seldom (29.60 ± 4.92 kg/m^2^ vs 27.16 ± 2.95 kg/m^2^), based on post hoc test. Patients never consuming oils had significantly higher BMI than patients consuming oils often (31.81 ± 4.09 kg/m^2^ vs 27.72 ± 3.92 kg/m^2^), based on post hoc test, table 17. Visualization of BMI by consumption groups, for food products differentiating it significantly, was presented in figure 10.

Table 17. Relationship of BMI (kg/m^2^) and consumption of selected food products

| **Variable** | **Never** | **Seldom** | **Often** | **p** |
| --- | --- | --- | --- | --- |
| White bread | 26.56 (25.83;28.64) | 28.28 (24.85;29.38) | 28.12 (25.91;31.11) | 0.746^4^ |
| Wholemeal bread | 27.53 ± 3.57 | 28.57 ± 4.37 | 28.45 ± 4.28 | 0.842 |
| Pastries | 27.47 ± 2.15 | 28.74 ± 4.69 | 28.28 ± 4.07 | 0.785 |
| Flour products | 29.54 ± 5.06 | 28.32 ± 4.41 | 28.25 ± 3.84 | 0.709 |
| Groats/rice/cereal | 28.16 ± 3.18 | 28.75 ± 4.51 | 28.15 ± 4.16 | 0.818 |
| Milk/milk beverages | 31.13 ± 6.86 | 27.97 ± 3.58 | 28.29 ± 4.08 | 0.524^3^ |
| White cheese | 27.32 ± 3.97 | 28.24 ± 4.22 | 28.77 ± 4.34 | 0.670 |
| Cheese/processed cheese | 29.06 ± 5.62 | 27.70 ± 3.69 | 29.24 ± 4.62 | 0.263 |
| Meet | 27.18 ± 3.62 | 29.77 ± 4.86 | 28.32 ± 4.19 | 0.481 |
| Poultry | - | 27.43 (25.45;30.34) | 28.12 (25.63;31.36) | 0.733^2^ |
| Offal | 28.78 ± 5.08 | 28.44 ± 3.78 | 25.65 ± 2.94 | 0.387 |
| Sausages | 26.89 ± 3.05 | 28.22 ± 4.12 | 29.44 ± 4.71 | 0.175 |
| Premium cold cuts | 29.82 ± 5.30 | 27.58 ± 3.26 | 28.71 ± 4.55 | 0.330 |
| Cold cuts | 28.83 ± 5.24 | 28.14 ± 4.40 | 28.54 ± 3.89 | 0.875 |
| Bacon/lard | 28.37 ± 4.59 | 28.46 ± 4.16 | 28.36 ± 4.25 | 0.995 |
| Meat canned food | 27.83 ± 4.15 | 28.51 ± 4.35 | 31.05 ± 2.32 | 0.359 |
| Fish canned food | 29.11 ± 4.49 | 28.58 ± 4.27 | 27.55 ± 4.00 | 0.509 |
| Fresh fish | 28.24 ± 5.74 | 27.58 ± 3.53 | 29.15 ± 4.55 | 0.263 |
| Eggs | 25.86 ± 3.15 | 28.44 ± 4.52 | 28.67 ± 4.14 | 0.373 |
| Butter | 28.14 ± 5.03 | 28.58 ± 4.46 | 28.41 ± 4.14 | 0.973 |
| Margarine | 29.60 ± 4.92^a^ | 27.16 ± 2.95^a^ | 28.99 ± 4.72 | **0.046^3^** |
| Oils | 31.81 ± 4.09^b^ | 28.80 ± 4.53 | 27.72 ± 3.92^b^ | **0.015** |
| Potatos | - | 27.77 (25.83;31.31) | 27.91 (25.36;30.43) | 0.600^2^ |
| Boiled vegetables | 23.82 ± 3.32 | 28.14 ± 3.80 | 28.81 ± 4.37 | 0.127 |
| Fresh vegetables | - | 28.36 (25.27;31.71) | 27.43 (25.41;30.07) | 0.531^2^ |
| Fruit | 26.50 (26.42;26.57) | 28.04 (25.56;29.62) | 28.06 (25.43;30.94) | 0.784^4^ |
| Sugar (for beverages) | 28.47 ± 4.08 | 27.74 ± 3.72 | 29.04 ± 4.96 | 0.571 |
| Jams | 29.79 ± 3.78 | 28.09 ± 4.25 | 28.24 ± 4.53 | 0.388 |
| Honey | 29.63 ± 3.95 | 28.05 ± 3.74 | 28.29 ± 4.59 | 0.527 |
| Sweet beverages | 29.26 ± 4.74 | 27.81 ± 3.92 | 29.49 ± 4.16 | 0.299 |
| Legumes products | 29.27 ± 3.98 | 28.36 ± 4.29 | 28.16 ± 4.36 | 0.832 |
| Beer | 28.93 ± 4.32 | 27.48 ± 3.67 | 30.03 ± 6.07 | 0.206 |
| Wine | 28.38 (25.47;31.89) | 27.24 (25.51;30.07) | - | 0.205^2^ |
| Vodka | 28.04 (25.47;31.89) | 27.68 (25.64;29.85) | - | 0.324^2^ |
| Coffee | 28.22 (26.43;32.64) | 24.72 (23.37;29.80) | 27.78 (25.69;30.67) | 0.498^4^ |
| Tea | 27.12 ± 2.90 | 28.57 ± 4.64 | 28.48 ± 4.32 | 0.785 |
| Vitamins | - | 28.05 ± 4.95 | 28.97 ± 4.17 | 0.362^1^ |

**Notes:** SD – standard deviation, Q1 – first quartile, Q3 – third quartile. Data presented as mean ± standard deviation or median (Q1;Q3), depending on normality of distribution. Comparisons performed with t-Student independent test^1^, Mann-Whitney U test^2^, Anova or Anova Welch^3^ analysis or Kruskal-Wallis test^4^. Post hoc evaluation run with Tukey test. a-b – adjusted p value produced with Tukey test (a: p = 0.042, b: p = 0.012).

Figure 10. BMI within consumption groups of margarine and oils (food products with significant relationship with BMI)

**Relationship between food products and sex**

Significant relationships between food products and sex were found for: sausages (p = 0.001), canned meat (p = 0.007), sugar for beverages (p = 0.049), beer (p < 0.001), vodka (p = 0.009) and tea (p = 0.010). Proportions of men not eating sausages and eating them seldom were lower than respective proportions of women (12.5%, n = 3 vs 19.0% and 25.0%, n = 6 vs 60.3%), while proportion of men consuming sausages often was higher than respective proportion of women (62.5% vs 20.7%). Canned meat was never consumer by 8.3% of men (n = 2) and 40.0% of women. Proportions of men eating canned meat seldom was higher than respective proportion of women (83.3% vs 56.7%) and proportion of men consuming canned meat often was higher than respective proportion of women (8.3%, n = 2 vs 3.3%, n = 2). Sugar for beverages was never used by 25.0% of men (n = 6) and 50.0% of women. Proportions of men and woman using sugar for beverages seldom was similar (29.2%, n = 7 and 28.3%, respectively), while higher proportion of men used it often compared to women (45.8% vs 21.7%). Proportion of men who resigned from beer consumption was lower than respective proportion of women (20.8%, n = 5 vs 65.0%). Seldom beer consumption was observed among 54.2% of men and 35.0% of women, while often beer consumption was declared by 25.0% of men (n = 6) and no women. Vodka was either not consumed or consumed seldom by both sexes. Proportion of patients not consuming vodka was lower within men, compared to women (29.2%, n = 7 vs 63.3%), while seldom consumption was noted in case of 70.8% of men and 36.7% of women. Majority of patients from both sex groups consumed tea often, however the proportion was higher in case of women (90.0% vs 65.2% among men). Seldom consumption of tea was observed more frequently among men compared to women (26.1%, n = 6 vs 5.0%, n = 3), while no tea consumption was met in case of 8.7% of men (n = 2) and 5.0% of women (n = 3), table 18.

Table 18. Correlation of sex and consumption of selected food products

| **Variable** | **Males** | | | **Females** | | | **p** |
| --- | --- | --- | --- | --- | --- | --- | --- |
|  | **Never** | **Seldom** | **Often** | **Never** | **Seldom** | **Often** |  |
| White bread | 3 (12.5) | 2 (8.3) | 19 (79.2) | 9 (15.3) | 8 (13.6) | 42 (71.2) | 0.855 |
| Wholemeal bread | 2 (8.3) | 12 (50.0) | 10 (41.7) | 5 (8.3) | 23 (38.3) | 32 (53.3) | 0.598^1^ |
| Pastries | 1 (4.2) | 9 (37.5) | 14 (58.3) | 4 (6.7) | 26 (43.3) | 30 (50.0) | 0.857 |
| Flour products | 3 (12.5) | 14 (58.3) | 7 (29.2) | 6 (10.0) | 28 (46.7) | 26 (43.3) | 0.486^1^ |
| Groats/rice/cereal | 2 (8.3) | 15 (62.5) | 7 (29.2) | 4 (6.7) | 23 (38.3) | 33 (55.0) | 0.080 |
| Milk/milk beverages | 2 (8.3) | 12 (50.0) | 10 (41.7) | 5 (8.3) | 16 (26.7) | 39 (65.0) | 0.111^1^ |
| White cheese | 3 (12.5) | 12 (50.0) | 9 (37.5) | 4 (6.7) | 24 (40.0) | 32 (53.3) | 0.371^1^ |
| Cheese/processed cheese | 2 (8.3) | 11 (45.8) | 11 (45.8) | 3 (5.0) | 33 (55.0) | 24 (40.0) | 0.677 |
| Meet | 1 (4.2) | 0 (0.0) | 23 (95.8) | 4 (6.7) | 10 (16.7) | 46 (76.7) | 0.074 |
| Poultry | 0 (0.0) | 12 (50.0) | 12 (50.0) | 1 (1.7) | 28 (46.7) | 31 (51.7) | > 0.999 |
| Offal | 5 (20.8) | 18 (75.0) | 1 (4.2) | 23 (38.3) | 34 (56.7) | 3 (5.0) | 0.276 |
| Sausages | 3 (12.5) | 6 (25.0) | 15 (62.5) | 11 (19.0) | 35 (60.3) | 12 (20.7) | **0.001^1^** |
| Premium cold cuts | 1 (4.2) | 7 (29.2) | 16 (66.7) | 7 (11.7) | 22 (36.7) | 31 (51.7) | 0.371^1^ |
| Cold cuts | 1 (4.2) | 10 (41.7) | 13 (54.2) | 10 (16.7) | 22 (36.7) | 28 (46.7) | 0.308^1^ |
| Bacon/lard | 4 (16.7) | 14 (58.3) | 6 (25.0) | 17 (28.3) | 35 (58.3) | 8 (13.3) | 0.311^1^ |
| Meat canned food | 2 (8.3) | 20 (83.3) | 2 (8.3) | 24 (40.0) | 34 (56.7) | 2 (3.3) | **0.007** |
| Fish canned food | 2 (8.3) | 15 (62.5) | 7 (29.2) | 14 (23.3) | 32 (53.3) | 14 (23.3) | 0.284^1^ |
| Fresh fish | 1 (4.2) | 11 (45.8) | 12 (50.0) | 4 (6.7) | 25 (41.7) | 31 (51.7) | 0.928 |
| Eggs | 1 (4.2) | 8 (33.3) | 15 (62.5) | 4 (6.7) | 21 (35.0) | 35 (58.3) | > 0.999 |
| Butter | 2 (8.3) | 8 (33.3) | 14 (58.3) | 5 (8.3) | 10 (16.7) | 45 (75.0) | 0.235^1^ |
| Margarine | 9 (37.5) | 8 (33.3) | 7 (29.2) | 24 (40.0) | 29 (48.3) | 7 (11.7) | 0.132^1^ |
| Oils | 5 (20.8) | 5 (20.8) | 14 (58.3) | 5 (8.3) | 12 (20.0) | 43 (71.7) | 0.259^1^ |
| Potatos | 0 (0.0) | 1 (4.3) | 22 (95.7) | 1 (1.7) | 11 (18.3) | 48 (80.0) | 0.199 |
| Boiled vegetables | 1 (4.2) | 10 (41.7) | 13 (54.2) | 2 (3.3) | 16 (26.7) | 42 (70.0) | 0.352 |
| Fresh vegetables | 0 (0.0) | 9 (37.5) | 15 (62.5) | 4 (6.7) | 17 (28.3) | 39 (65.0) | 0.453 |
| Fruit | 1 (4.2) | 1 (4.2) | 22 (91.7) | 1 (1.7) | 6 (10.0) | 53 (88.3) | 0.449 |
| Sugar (for beverages) | 6 (25.0) | 7 (29.2) | 11 (45.8) | 30 (50.0) | 17 (28.3) | 13 (21.7) | **0.049^1^** |
| Jams | 4 (16.7) | 13 (54.2) | 7 (29.2) | 11 (18.3) | 38 (63.3) | 11 (18.3) | 0.548^1^ |
| Honey | 5 (20.8) | 11 (45.8) | 8 (33.3) | 8 (13.3) | 15 (25.0) | 37 (61.7) | 0.061^1^ |
| Sweet beverages | 7 (30.4) | 14 (60.9) | 2 (8.7) | 23 (38.3) | 35 (58.3) | 2 (3.3) | 0.548 |
| Legumes products | 0 (0.0) | 22 (91.7) | 2 (8.3) | 8 (13.3) | 44 (73.3) | 8 (13.3) | 0.125 |
| Beer | 5 (20.8) | 13 (54.2) | 6 (25.0) | 39 (65.0) | 21 (35.0) | 0 (0.0) | **< 0.001** |
| Wine | 8 (34.8) | 15 (65.2) | 0 (0.0) | 33 (55.0) | 27 (45.0) | 0 (0.0) | 0.160^1^ |
| Vodka | 7 (29.2) | 17 (70.8) | 0 (0.0) | 38 (63.3) | 22 (36.7) | 0 (0.0) | **0.009^1^** |
| Coffee | 2 (8.7) | 1 (4.3) | 20 (87.0) | 8 (13.3) | 5 (8.3) | 47 (78.3) | 0.724 |
| Tea | 2 (8.7) | 6 (26.1) | 15 (65.2) | 3 (5.0) | 3 (5.0) | 54 (90.0) | **0.010** |
| Vitamins | 0 (0.0) | 8 (28.6) | 20 (71.4) | 0 (0.0) | 20 (33.3) | 40 (66.7) | 0.841^1^ |

**Notes:** Data presented as n (% of females or males). Comparisons performed with Pearson chi-square test^1^ or Fisher exact test.
